# Polyacrylonitrile Passivation for Enhancing the Optoelectronic Switching Performance of Halide Perovskite Memristor for Image Boolean Logic Applications

**DOI:** 10.3390/nano13152174

**Published:** 2023-07-26

**Authors:** Xiaohan Zhang, Xiaoning Zhao, Zhongqiang Wang

**Affiliations:** Key Laboratory of UV-Emitting Materials and Technology of Ministry of Education, Northeast Normal University, Changchun 130024, China

**Keywords:** organic–inorganic halide perovskites, polyacrylonitrile, ion migration, optoelectronic memristor, image Boolean operations

## Abstract

For the CH_3_NH_3_PbI_3_-based optoelectronic memristor, the high ion-migration randomness induces high fluctuation in the resistive switching (RS) parameters. Grain boundaries (GBs) are well known as the ion-migration sites due to their low energy barrier. Herein, a polyacrylonitrile (PAN) passivation method is developed to reduce GBs of the CH_3_NH_3_PbI_3_ film and improve the switching uniformity of the memristor. The crystal grain size of CH_3_NH_3_PbI_3_ increases with the addition of PAN, and the corresponding number of GBs is consequently reduced. The fluctuations of the RS parameters of the memristor device are significantly reduced. With the memristor, nonvolatile image sensing, image memory, and image Boolean operations are demonstrated. This work proposes a strategy for developing high-performance CH_3_NH_3_PbI_3_ optoelectronic memristors.

## 1. Introduction

In the past decade, organic–inorganic halide perovskites (OIHPs) have emerged as desirable materials for a wide range of optoelectronic applications, including solar cells [1,2], light-emitting diodes [3,4], photodetectors, etc. [5,6]. OIHPs possess a soft crystal structure, which facilitates the migration of low activation energy defects (such as vacancies, interstitials or anti-site substitutions) under the drive of electric field and/or light [7,8]. Hysteresis phenomena in current–voltage (I–V) measurements caused by ion migration have been observed in solar cells, which also presents significant potential for memristive applications [9,10]. The OIHP-based memristors have great potential for next-generation memory and various optoelectronic applications.

Generally, the formation/rupture of conductive filaments (CFs) in OIHP resulting from ions’ migration is the main working mechanism of memristor devices [11,12]. In addition to electric field, light irradiation can also be involved in the halide ion-migration kinetics [13,14]. However, uncontrollable ion transport or diffusion leads to disorderly growth and breakage of the CFs, which eventually leads to inhomogeneity and instability in the resistive switching (RS) voltage and resistance states. Therefore, it is crucial to explore effective strategies to guide the formation and rupture of CFs to achieve a highly uniform optoelectronic switching behavior. In OIHP, the grain boundaries (GBs) provide a shortcut for ion migration because the activation energy of halide ions is minimal at the GBs [15,16]. Therefore, controlling the number of GBs could reduce the CFs’ randomness for enhancing the switching performance of OIHP-based memristors.

Recently, the phenomenon of light-driven manipulation of the memristor state has been reported in CH_3_NH_3_PbI_3_ [7], HfO_2_ [17], ZnO [18], and other materials. Hence, the application range of memristor devices can be expanded by relying on the optical modulation characteristics. Furthermore, the phenomenon of light-driven manipulation of the memristor state may be utilized in image sensing and logic operations. Zhao et al. obtained the image sensing functions based on CsPbBr_x_I_3−x_ optoelectronic memristors, whose resistance state can be modulated by photo-induced halide vacancies-assisted CFs breakage [19]. Chai et al. demonstrated the “OR” and “AND” optoelectronic logic operations with a CH_3_NH_3_PbI_3−x_Cl_x_ optoelectronic memristor, which was attributed to the modulation of the CH_3_NH_3_PbI_3−x_Cl_x_/Au interface barrier by light [20]. However, to the best of our knowledge, there is no report on image Boolean logic operations with CH_3_NH_3_PbI_3_ memristors.

In this study, polyacrylonitrile (PAN) is introduced into the precursor solution to modify the nucleation and crystal growth process of CH_3_NH_3_PbI_3_ films for optoelectronic memristive applications. The influence of PAN concentration on the grain size and switching performance of CH_3_NH_3_PbI_3_ memristors is systematically investigated. The CH_3_NH_3_PbI_3_ memristors with uniform switching characteristics are obtained and photo-induced switching behaviors have been confirmed. Through the synergy of electrical and optical stimuli, nonvolatile image sensing and image Boolean operations are demonstrated.

## 2. Materials and Methods

### 2.1. Sample Preparation

The fluorine-doped tin oxide (FTO) glasses substrates were cleaned with trichloroethene, acetone, ethanol, and deionized water, successively. The perovskite film on the FTO substrates was prepared by a one-step method. Specifically, the perovskite precursor solution was prepared by mixing 159 mg CH3NH3I, 461 mg PbI2, and different concentrations (0, 1, 2, 3 and 4 mg/mL) of the PAN additives in 1 mL N, N-Dimethylformamide (DMF), and stirring under ambient temperature for 2 h. The resulting precursor solution was spin-coated on FTO substrate at a speed of 4000 rpm for 20 s, and antisolvent ethyl acetate was quickly dropped onto the center of the substrate during spin-coating. After the spin coating was completed, all samples were annealed on a hot stage at 90 °C for 30 min to obtain the CH_3_NH_3_PbI_3_ films. Finally, Au top electrodes with a side length of 300 μm were deposited by thermal evaporation through a shadow mask in square patterns.

### 2.2. Characterization

The I–V characteristics of the Au/CH3NH3PbI3/FTO memristor were measured using a semiconductor parameter analyzer (2636A, Keithley, OH, USA) at room temperature, and the bottom electrode (FTO) was grounded. The morphology of CH3NH3PbI3 films were measured by a field emission scanning electron microscopy (SEM) (SU8010, Hitachi, Tokyo, Japan). The X-ray diffraction (XRD) measurements were conducted using an X-ray diffractometer (D/max-2500, Rigaku, Tokyo, Japan), and the XRD data were recorded at room temperature. The transient-state photoluminescence (PL) was measured by a spectrometer (LavRAM HR Evollution, Horiba, Kyoto, Japan), and the excitation wavelengths’ center was 488 nm. The optoelectronic measurements were performed using a xenon lamp (LA-410UV, Hayashi, Tokyo, Japan).

## 3. Results

**Figure 1:**
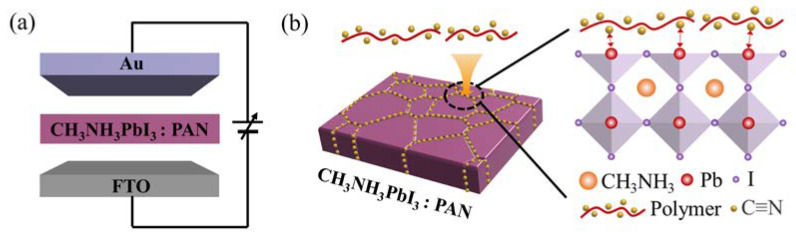
(**a**) Schematic illustration of an Au/CH_3_NH_3_PbI_3_/FTO memristor device. (**b**) Schematic illustration of the PAN-passivated CH_3_NH_3_PbI_3_ films.

In the current study, a PAN passivation method was developed to improve the switching uniformity of the CH3NH3PbI3 memristor. The CH3NH3PbI3 films with different concentrations of PAN were prepared for memristors. Schematic illustrations of the memristor device and the PAN-passivated CH3NH3PbI3 films are described in Figure 1a,b, respectively. Here, the PAN was mainly distributed at the GBs of the CH3NH3PbI3 films, acting as “impurities”, because the GBs are the preferred sites for impurity segregation [21]. The PAN with cyano-groups can passivate the uncoordinated Pb^2+^ and modify the nucleation and crystal growth processes of the perovskite films to increase the grain size [22,23]. 

**Figure 2:**
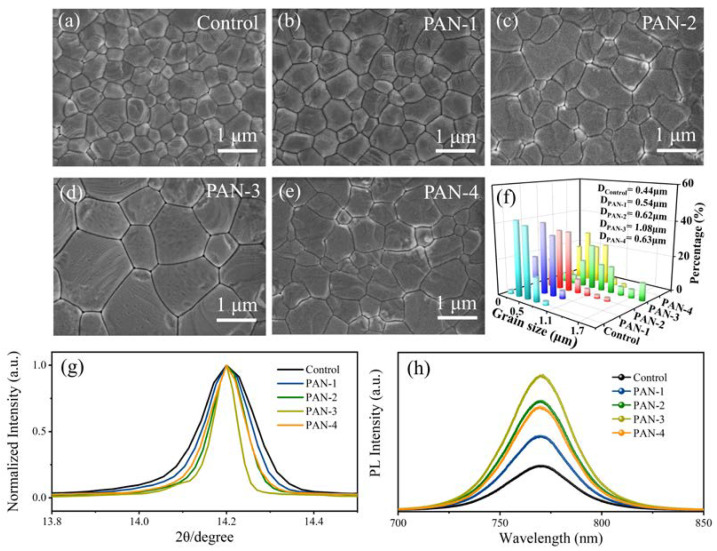
Top-view SEM images of PAN passivation CH_3_NH_3_PbI_3_ films with different concentrations of PAN additives: (**a**) Control, (**b**) PAN-1, (**c**) PAN-2, (**d**) PAN-3, and (**e**) PAN-4. (**f**) Statistics of the averaged grain sizes of the CH_3_NH_3_PbI_3_ films under all the above conditions. (**g**) The normalized intensities of (110) diffraction peaks in the XRD patterns and (**h**) PL spectra of CH_3_NH_3_PbI_3_ films with different concentrations of PAN additives.

In order to investigate the correlation between the PAN additives’ concentration and grain size of the CH_3_NH_3_PbI_3_ perovskite films, we obtained the surface morphology SEM image of CH_3_NH_3_PbI_3_ perovskite films with 0–4 mg/mL of PAN additives’ concentration. Figure 2a–e illustrates the top-view SEM images of the CH_3_NH_3_PbI_3_ films with different concentrations of PAN (0 mg/mL (Control), 1 mg/mL (PAN-1), 2 mg/mL (PAN-2), 3 mg/mL (PAN-3), and 4 mg/mL (PAN-4), respectively). The CH_3_NH_3_PbI_3_ films exhibited larger grain size after PAN passivation compared to the control films without PAN passivation. With a modest concentration of PAN additives (1–3 mg/mL), the mean grain size of the CH_3_NH_3_PbI_3_ films increased from ~0.54 μm (PAN-1) and ~0.62 μm (PAN-2) to ~1.08 μm (PAN-3). We attribute the larger grain size to the PAN additives, which can provide nucleation sites to guide the preferential orientation growth of crystals and thereby increases the grain size [22,23]. It is worth noting that the sample PAN-3 presented the largest grain size (Figure 2d), which means that PAN-3 had the minimum number of GBs. To confirm the reproducibility of this method, three samples with the same amounts of reagents (PAN-3) were prepared. The top-view SEM image and the distribution statistics of the grain size are shown in Figure S1. Sample 1, sample 2, and sample 3 exhibited similar morphology and grain size, confirming the reproducibility of this method. However, a higher concentration (4 mg/mL) of PAN additives provided a surplus of nucleation sites for CH_3_NH_3_PbI_3_ films, thereby, led to a decrease in grain size (~0.63 μm) (Figure 2e). Figure 2f shows the distribution of the grain size and statistics of mean grain size in the CH_3_NH_3_PbI_3_ films prepared with different concentrations of PAN additives, which further supports that the largest grain size of CH_3_NH_3_PbI_3_ films was achieved at the PAN additives’ concentration of 3 mg/mL. 

The effect of PAN additives on the crystallization of the CH_3_NH_3_PbI_3_ films was studied by XRD (Figure S2 and Figure 2g). XRD patterns of CH_3_NH_3_PbI_3_ films with different concentrations of PAN additives are shown in Figure S2. The XRD patterns of all CH_3_NH_3_PbI_3_ films samples revealed peaks at 14.2° and 28.5°, corresponding to the reflections from the (110) and (220) planes [24], respectively. The XRD patterns meant that the PAN additives had no effect on the crystal structure and orientation of CH_3_NH_3_PbI_3_. As shown in Figure 2g, by normalizing the (110) planes of the XRD patterns, the sample with the addition of PAN exhibited the narrower full-width at half-maximum, indicating the formation of larger grain size in the CH_3_NH_3_PbI_3_ film [25]. Moreover, we measured the PL spectra of the CH_3_NH_3_PbI_3_ films with different concentrations of PAN additives. Figure 2h reveals the tendency of PL intensity to remain fixed at ~765 nm for all the CH_3_NH_3_PbI_3_ film samples. We found that the PL intensities of the CH_3_NH_3_PbI_3_ films with PAN additives were higher than that of the control film, indicating that carrier recombination in the CH_3_NH_3_PbI_3_ layer was significantly inhibited, which means that the number of GBs of CH_3_NH_3_PbI_3_ films was reduced (GBs are the main nonradiative recombination centers) [26]. In addition, the UV–vis absorption spectra of the CH_3_NH_3_PbI_3_ films with different concentrations of PAN additives were also measured, as presented in Figure S3. When PAN concentration increased from 0 to 3 mg/mL, the light absorption intensities of the CH_3_NH_3_PbI_3_ films were enhanced slightly. By further increasing the PAN concentration, the light absorption intensity was reduced. The change in absorption would result from the combined effect of better crystallinity and increased grain size [27,28]. Based on the XRD, PL, and UV–vis absorption test results, it can be inferred that the PAN-3 sample exhibited the largest grain size, with a minimum number of GBs.

**Figure 3:**
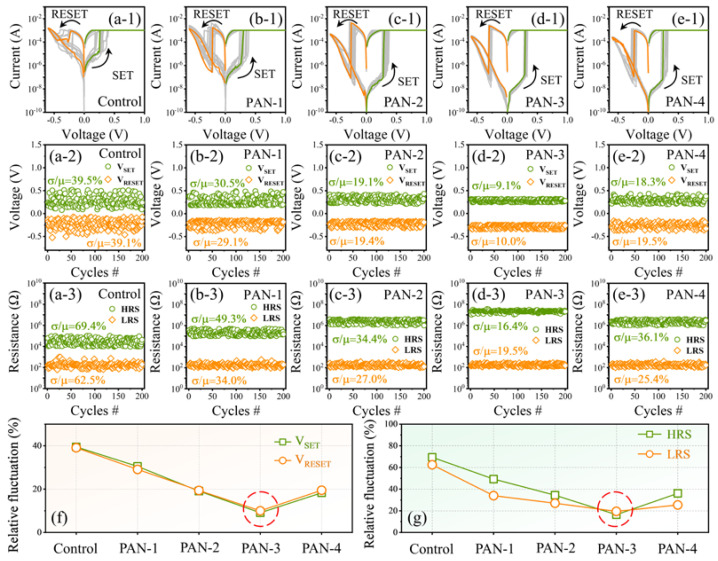
RS performance of the Au/CH_3_NH_3_PbI_3_/FTO memristors with five different concentrations of PAN additives. (**a-1**–**e-1**) Typical I−V curves of the devices. Statistic (**a-2**–**e-2**) HRS/LRS and (**a-3**–**e-3**) V_SET_/V_RESET_ of the memristor devices over 200 cycles. (**f**,**g**) The relative fluctuations in V_SET_/V_RESET_ and HRS/LRS of the memristor devices.

To study the effect of crystal grain size on the RS performance of the CH_3_NH_3_PbI_3_ memristor, the electrical characteristics of the memristor devices with different concentrations of PAN additives were systematically investigated (Figure 3). As shown in Figure 3(a-1)–(e-1), I–V characteristics curves of ten consecutive RS cycles in the memristor with five types of CH_3_NH_3_PbI_3_ films were measured, and the measurements were carried out under dark conditions. The voltage applied on the Au top electrode was swept by a DC bias voltage as follows: 0 V→1 V→0 V→−0.6 V→0 V, and the limiting current was set as 1 mA to prevent the hard breakdown of the device. The five types of CH_3_NH_3_PbI_3_ memristors presented the typical bipolar RS behaviors. When positive voltage was swept to a SET value (V_SET_), the resistance of the device was changed from the high-resistance state (HRS) to the low-resistance state (LRS). When negative voltage was swept to a RESET value (V_RESET_), the resistance state was switched from LRS to the HRS. In general, for a fresh CH_3_NH_3_PbI_3_ memristor device, the electroforming process is required to activate the subsequent RS behavior [29]. The high voltage used in the electroforming process can generate defects in the CH_3_NH_3_PbI_3_ layer. For the CH_3_NH_3_PbI_3_ materials, iodide ions are the main migration defect due to the low migration barrier [7]. However, the five types of CH_3_NH_3_PbI_3_ memristors prepared in this work did not require the electroforming process to activate, which may be due to the production of sufficient iodide defects during the CH_3_NH_3_PbI_3_ films’ deposition process.

Figure 3(a-2)–(e-2) and Figure 3(a-3)–(e-3) show the relative distributions of V_SET_/V_RESET_ and HRS/LRS, respectively, in the five types CH_3_NH_3_PbI_3_ memristors for 200 consecutive RS cycles. In previous reports, the relative fluctuation defined by σ/µ (σ is the standard deviation, µ is the mean value) is generally used to evaluate the fluctuation of the RS parameter of the memristor [30]. Compared with the CH_3_NH_3_PbI_3_ memristor devices without the PAN additives, the RS parameter fluctuations in the memristors with the PAN additives were significantly reduced. When the concentration of PAN increased from 0 to 3 mg/mL, the relative fluctuations of HRS/LRS and V_SET_/V_RESET_ were reduced from 69.4%/62.5% and 39.5%/39.1% to 16.4%/19.5% and 9.1%/10.0%, respectively. Further increasing the concentration of PAN additives to 4 mg/mL impeded the optimization of the relative fluctuations in RS parameters. The relative fluctuation coefficients of V_SET_/V_RESET_ and HRS/LRS during 200 consecutive RS cycles in the five types of CH_3_NH_3_PbI_3_ memristors are more clearly demonstrated in Figure 3f,g. The relative fluctuation coefficients of V_SET_/V_RESET_ and HRS/LRS show a similar variation trend to the number of GBs in CH_3_NH_3_PbI_3_ films (as shown in Figure 2a–e), first reducing and then increasing with the change in PAN concentration. To sum up, the PAN-3 memristor exhibits minimal RS parameter fluctuations, which could benefit from the reduction in the number of GBs in the CH3NH3PbI3 films. 

Complex defects such as vacancies, interstitials, and anti-site occupations have been reported to exist in the solution-proceeding polycrystalline CH_3_NH_3_PbI_3_ films [31]. Among them, the iodide ions have a low activation energy and easily migrate under the action of electric field [7]. Thus, in previously reported studies of CH_3_NH_3_PbI_3_-based memristors, the RS mechanism is usually attributed to the formation and rupture of CFs resulting from iodide ions migration [10]. In addition, it has been reported that the activation energy of iodide ions’ migration at the GBs may be lower than that in the grain interior [32]. Based on the above reasons, it is reasonable to believe that the iodide vacancies-assisted CFs are mainly formed at GBs. Thus, the decrease in GBs can reduce the migration paths of iodide ions, a phenomenon which is responsible for the reduction in the randomness of CFs and the improved uniformity of RS parameters.

**Figure 4:**
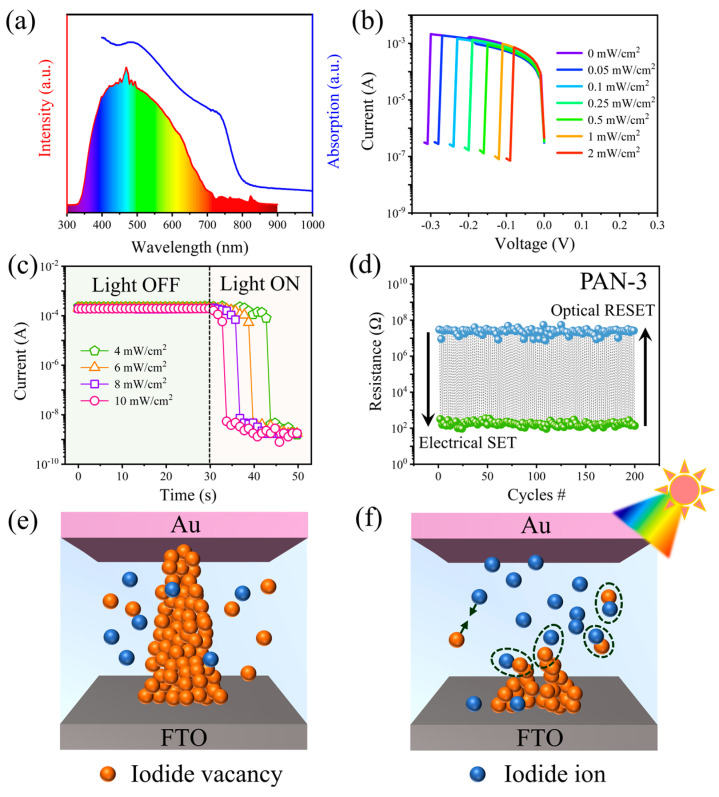
(**a**) Absorption spectra of the CH_3_NH_3_PbI_3_ film (PAN-3) and the spectra of visible light source. (**b**) The RESET processes of the memristor device under different light intensities. (**c**) Evolution of LRS of the CH_3_NH_3_PbI_3_ memristor device under light irradiation. (**d**) Cyclic electrical SET and optical RESET of the CH_3_NH_3_PbI_3_ memristor device. Schematic diagrams of (**e**) the electrical SET and (**f**) the optical RESET mechanism.

According to previous reports, the light can also induce the redistribution of iodide vacancies, and the recombination process of iodide vacancies and iodide ions in the film are then promoted [7]. As reported by Lu et al., light illumination can facilitate the breakage of CFs in the CH_3_NH_3_PbI_3_ films [7]. We studied the optoelectronic characteristics of the optimized PAN-3 memristor device during the RESET process using visible light with different intensities. Herein, the visible light is obtained with a xenon lamp source (the spectrum is shown in Figure 4a). The PAN-3 sample films shows the optical absorption in the visible light range (Figure 4a). As shown in Figure 4b, we can see that V_RESET_ decreased significantly after visible light stimulation. The V_RESET_ required to switch the memristor device steadily decreased from −0.31 V to −0.09 V when the light intensity was increased from 0 to 2 mW/cm^2^. We also studied the effect of light wavelengths on the RESET process of the optoelectronic memristor. Three different wavelengths, centered at 415 nm (blue light), 543 nm (green light), and 620 nm (red light), were obtained by applying different bandpass filters to the visible light source, and their spectra can be found in Figure S4a. It is interesting that the V_RESET_ reduced from −0.31 V to 0.15 V, 0.19 V, and 0.24 V after the same intensity irradiation processes of blue light, green light, and red light (Figure S4b), respectively. We can find that the dependence tendency between V_RESET_ and the light wavelength is consistent with the light absorption intensity of the PAN-3 sample films (Figure 4a), that is, the shorter wavelength light is more effective in reducing the V_RESET_; such a feather can be developed for distinguishing colors. On the other hand, further measurements showed that, after programming the optoelectronic memristors to LRS in the dark, high-intensity visible light irradiation (4–10 mW/cm^2^) can result in rapid failure of LRS retention (Figure 4c). In this case, the optoelectronic memristor can switch from the LRS to the HRS with light illumination. It is worth noting that the above results support the ability of applied electric field and light irradiation to control ion migration process in the CH_3_NH_3_PbI_3_ films, which can be utilized to realize photocoupled memristor devices. As shown in Figure 4d, reproductive switching can be achieved by alternating electrical and optical stimuli. The visible light intensity of 10 mW/cm^2^ was used in the subsequent electrical SET and optical RESET cycle tests. As shown in Figure 4d, the optimized PAN-3 memristor device was tested over 100 optoelectronic cycles, and the device showed good repeatability and non-volatile performance. 

The above results indicate that the iodide vacancies-assisted CFs may rupture when the memristor is stimulated by light. This supposition is also supported by previous research. Angelis et al. proposed a model in which light illumination induced the annihilation of iodide ion/iodide vacancy Frenkel pairs in the CH_3_NH_3_PbI_3_ films by combining experimental and theoretical studies [14]. Therefore, the RS mechanism of the CH_3_NH_3_PbI_3_ memristor device in the process of electrical SET and optical RESET can be explained by the following model. In the initial state, a small number of iodide ions or iodide vacancies defects are randomly present in CH_3_NH_3_PbI_3_ films. As the voltage is applied, the iodide ions migrate towards the top electrode under the action of electric field and form iodide vacancies-assisted CFs (Figure 4e). When the CH_3_NH_3_PbI_3_ memristor is stimulated by light, the recombination process of iodide vacancies and iodide ions in the film is enhanced, resulting in the breakdown of CFs (Figure 4f).

**Figure 5:**
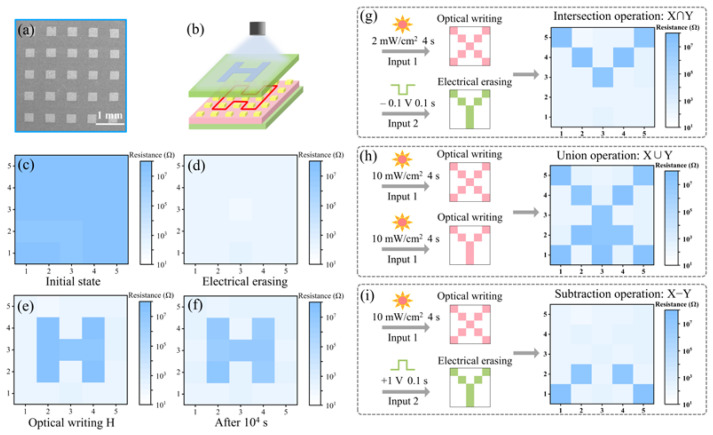
(**a**) SEM image of the 5 × 5 memristor device array. (**b**) Schematic diagram of the image written operation. (**c**) The initial and (**d**) erasing state of the memristor array. (**e**) The “H” image written and (**f**) the non-volatile “H” image storage. (**g**–**i**) Image Boolean logic operation of the memristor device array.

Based on the optoelectronic switching characteristics of the optimized PAN-3 memristor device, we construct an optoelectronic memristor array (5 × 5, Figure 5a) to demonstrate the non-volatile image storage and image Boolean logic function. Visible light illumination on the device was controlled by a specific mask (Figure 5b). In this part of the work, the HRS and LRS states were defined as logical “1” and logical “0”, respectively. The operation mode of the optoelectronic memristor array was “electrical erase, optical write”. The initial CH_3_NH_3_PbI_3_ memristor devices were all in HRS and were pre-switched to LRS by applying a voltage pulse (1 V, 0.1 s) prior to the write operation (Figure 5c,d). The stability of resistance state is crucial for the memristor device to perform logical operations. In order to more intuitively evaluate the RS uniformity of the optoelectronic memristor array, the color maps of HRS (after optical write) and LRS (after electrical erase) for devices are shown in Figure S5a,c. The corresponding histogram distributions results are summarized in Figure S5b,d, respectively. These show that all devices could operate normally and the resistance state was uniform. As shown in Figure 5e, light stimulus (10 mW/cm^2^, 4 s) was applied to the array with an “H”-type metal mask, and the image of “H” was written to achieve image storage. The “H” image was still visible after 10^4^ s (Figure 5f), indicating non-volatile optoelectronic information storage.

The optoelectronic switching characteristics also enable the devices to perform image logical operations. Boolean operation is a key logic algorithm widely used in image processing [33]. Through Boolean logic operation, “X”- and “Y”-shape images can be transformed into a new image “N”, and the operational rule can be categorized into intersection (N = X ∩ Y), union (N = X ∪ Y), and subtraction (N = X − Y) [34,35,36]:

(1) $$\{\begin{array}{c} \;X\;\cap^{\;}\;Y=\{N|N\in X\;\mathrm{and}\;N\in Y\\ \;X\;\cup^{\;}\;Y=\{N|N\in X\;\mathrm{or}\;N\in Y\;\;\;\\ \;X-Y=\{N|N\in X\;\mathrm{and}\;N\in \;\tilde{Y} \end{array}$$

Herein, image Boolean operations (intersection, union, and subtraction) were successfully implemented with the CH_3_NH_3_PbI_3_ memristor. All memristors were pre-switched to LRS by applying an electrical pulse (1 V, 0.1 s), and a read electrical pulse (0.1 V, 0.1 s) was applied after each logic operation. For the “intersection” operation, the implementation process was as shown in Figure 5g. The “X” image was input with optical pulse (2 mW/cm^2^, 4 s) as input and the “Y” image was input with electrical pulse (−0.1 V, 0.1 s). Only the memristor devices subjected to both optical stimuli and electrical stimuli could be switched from LRS to HRS. Therefore, “V”-shape output image could be obtained. For the “union” operation, the implementation process was as shown in Figure 5h. Images “X” and “Y” were input with optical pulses (10 mW/cm^2^, 4 s). All memristors subjected to optical stimuli could be switched from LRS to HRS. The implementation of “subtraction” operation was as shown in Figure 5i. First, the optical pulse (10 mW/cm^2^, 4 s) was used as the input of the “X” image. Then, the “Y” shaped electrical image was input with an electrical pulse (1 V, 0.1 s). Only the resistance state of the (X − Y)-shaped region was HRS, while the other regions were LRS, indicating that the “subtraction” operation of Boolean logic had been implemented. In this part, the stability of switching voltage is crucial for the device to perform logical operations. For example, if the optoelectronic memristor with large switching voltage fluctuation (Control, 0 mg/mL PAN) is used to implement the intersection logic operation, some memristor devices would not be switched as desired. As shown in Figure S6, under the combined stimulation of −0.1 V voltage and 2 mW/cm^2^ light, a portion of the optoelectronic memristor does not switch from LRS to HRS, and the logic operation cannot be performed successfully. Hence, the structure modification to improve the switching uniformity of the memristor is of great importance for optoelectronic image-processing.

The interaction of light with the memristor device allows the information stored in the memristor to be controlled remotely. In addition, light-driven manipulation of the memristor state can also realize more complex and higher-order logic operations. In this work, we used binary input values of light intensity and electric voltage to achieve image Boolean logic operations. In fact, perovskite optoelectronic memristors can also achieve continuous changing of resistance states by modulating the stimuli (voltage pulses) [37]. In the future, we can also regulate light and voltage and monitor the input–output relationship in a continuous way, implementing fuzzy logic operations based on multi-valued logic [38,39].

## 4. Conclusions

In summary, the PAN passivation method was introduced to reduce the number of GBs in CH_3_NH_3_PbI_3_ films and minimize the CF randomness. As a result, the fluctuations in RS parameters (HRS/LRS and V_SET_/V_RESET_) in the optoelectronic CH_3_NH_3_PbI_3_ memristor devices was significantly reduced. In addition, the CH_3_NH_3_PbI_3_ memristor could be modulated, not only by electrical signals, but also by optical pulses. The unique optoelectronic characteristics enabled the device to achieve image sensing and image Boolean logic operations. This work provides an effective method for developing high-performance CH_3_NH_3_PbI_3_ optoelectronic memristors. 

## Data Availability

All data generated and analyzed during this study are included in this article.

## Supplementary Materials

The following supporting information can be downloaded at: https://www.mdpi.com/article/10.3390/nano13152174/s1, Figure S1: (a–c) The SEM images of three CH_3_NH_3_PbI_3_ films prepared with a same PAN concentration (3 mg/mL). (d–f) The distribution statistics of the grain size of sample 1, sample 2 and sample 3. Figure S2: XRD patterns of the CH_3_NH_3_PbI_3_ films with different concentrations of PAN additives. Figure S3: The optical absorption spectra of the CH_3_NH_3_PbI_3_ films with different concentrations of PAN additives. Figure S4: (a) The spectra distribution of light obtained by applying different bandpass filters to the visible light source. (b) The switching curve of the device with different light wavelength and the density is fixed at 1 mW/cm^2^. Figure S5: (a,c) Color maps of HRS and LRS of optoelectronic memristor array. (b,d) Histogram distributions of HRS and LRS in 36 optoelectronic memristor. Figure S6: Boolean intersection operations performed with the optoelectronic memristor (Control, 0 mg/mL PAN).
