# Supplementary material for: Polyacrylonitrile Passivation for Enhancing the Optoelectronic Switching Performance of Halide Perovskite Memristor for Image Boolean Logic Applications
Source: Nanomaterials (Basel). 2023 Jul 26;13(15):2174. doi: 10.3390/nano13152174 (PMC10421001; doi:10.3390/nano13152174)
Supplement: Supplementary file 1 [file nanomaterials-13-02174-s001.zip › nanomaterials-2502395-supplementary.pdf]

Supporting Information

Polyacrylonitrile Passivation for Enhancing the Optoelectronic Switching Performance of Halide Perovskite Memristor for Image Boolean Logic Applications

Xiaohan Zhang [1], Xiaoning Zhao [1,*] and Zhongqiang Wang [1,*]

[1] Key Laboratory of UV-Emitting Materials and Technology of Ministry of Education, Northeast Normal University, Changchun 130024, China

* Correspondence: zhaoxn430@nenu.edu.cn (X.Z.); wangzq752@nenu.edu.cn (Z.W.)

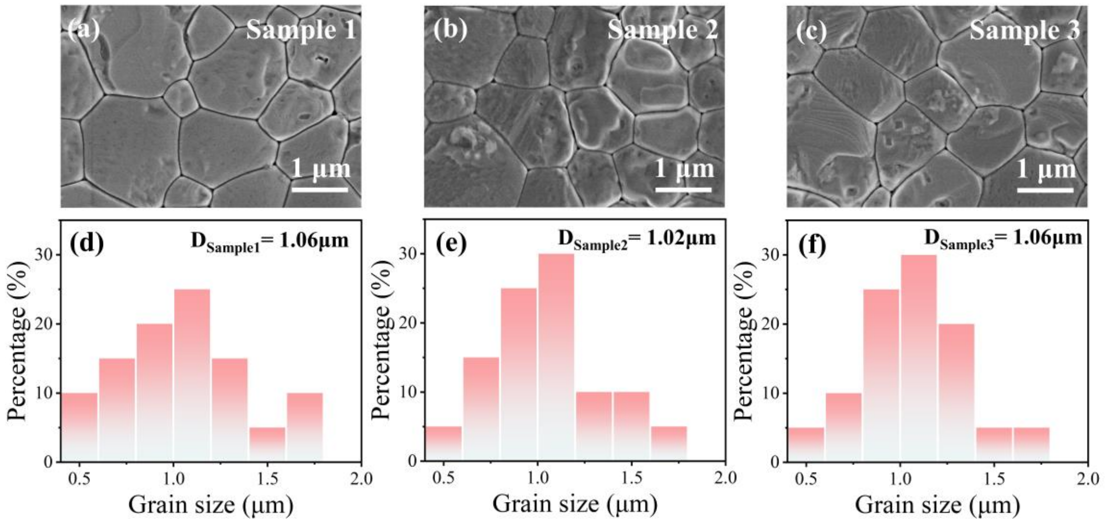

Figure S1. (a-c) The SEM images of three $CH_3NH_3PbI_3$ films prepared with a same PAN concentration (3 mg/mL). (d-f) The distribution statistics of the grain size of sample 1, sample 2 and sample 3.

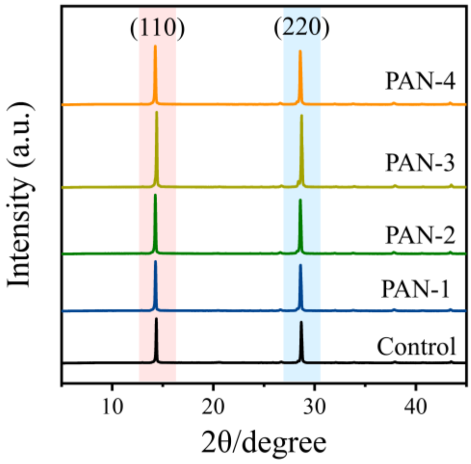

Figure S2. XRD patterns of the $CH_3NH_3PbI_3$ films with different concentrations of PAN additives.

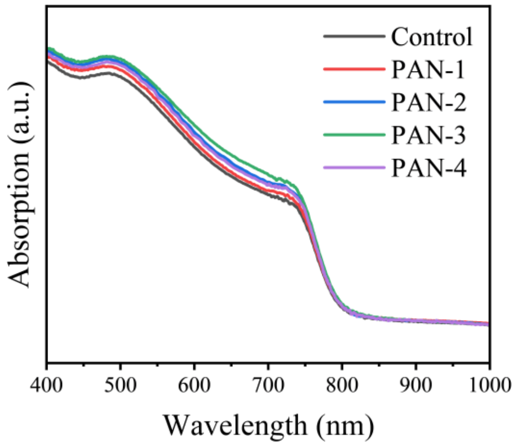

Figure S3. The optical absorption spectra of the $CH_3NH_3PbI_3$ films with different concentrations of PAN additives.

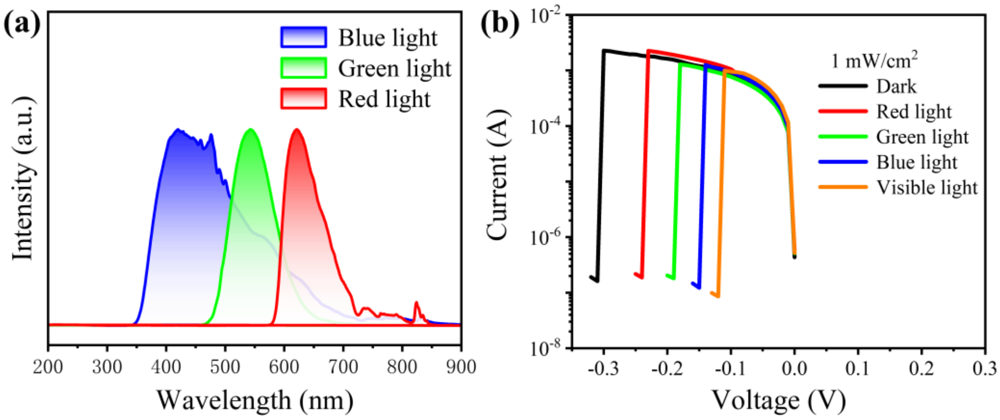

Figure S4. (a) The spectra distribution of light obtained by applying different bandpass filters to the visible light source. (b) The switching curve of the device with different light wavelength and the density is fixed at 1 mW/cm^2.

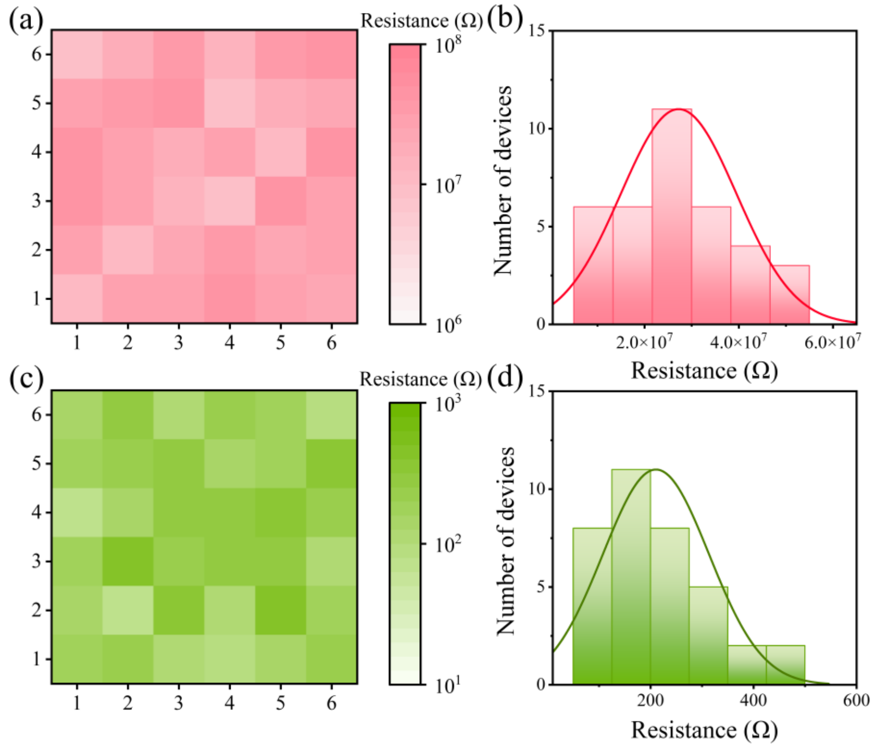

Figure S5. (a, c) Color maps of HRS and LRS of optoelectronic memristor array. (b, d) Histogram distributions of HRS and LRS in 36 optoelectronic memristor.

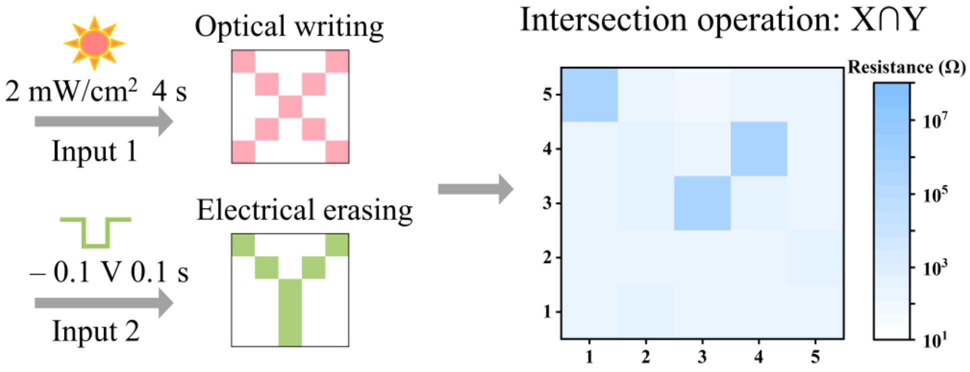

Figure S6. Boolean intersection operations performed with the optoelectronic memristor (Control, 0 mg/mL PAN).
